# Cognitive and anti-inflammatory effects of *Palmaria palmata* in a schizophrenia mouse model: insights into CREB signaling, Iba-1 expression, and CD4+ cell modulation

**DOI:** 10.3389/fnins.2025.1551764

**Published:** 2025-06-04

**Authors:** Shimaa Mohammad Yousof, Badrah S. Alghamdi, Thamer Alqurashi, Mohammad Zubair Alam, Reham Tash, Noha M. Abd El-Fadeal, Samy A. Abusikkien, Lamis Kaddam

**Affiliations:** ^1^Department of Physiology, Faculty of Medicine, King Abdulaziz University, Jeddah, Saudi Arabia; ^2^Department of Physiology, Faculty of Medicine, Suez Canal University, Ismailia, Egypt; ^3^Neuroscience and Geroscience Unit, King Fahad Medical Research Centre, King Abdulaziz University, Jeddah, Saudi Arabia; ^4^Neuroscience Unit, Department of Physiology, Faculty of Medicine, King Abdulaziz University, Jeddah, Saudi Arabia; ^5^Pharmacology Department, King Abdulaziz University, Rabigh, Saudi Arabia; ^6^Department of Medical Laboratory Technology, Faculty of Applied Medical Sciences, King Abdulaziz University, Jeddah, Saudi Arabia; ^7^Department of Anatomy, Faculty of Medicine, King Abdulaziz University, Rabigh, Saudi Arabia; ^8^Department of Anatomy, Faculty of Medicine, Ain Shams University, Cairo, Egypt; ^9^Medical Biochemistry and Molecular Biology Department, Faculty of Medicine, Suez Canal University, Ismailia, Egypt; ^10^Biochemistry Department, Ibn Sina National College for Medical Studies, Jeddah, Saudi Arabia; ^11^Physiology Department, Faculty of Medicine, Alneelain University, Khartoum, Sudan

**Keywords:** schizophrenia, object recognition memory, anxiety, red marine algae, *Palmaria palmata*, zero maze, marble burying test, Y maze

## Abstract

**Background:**

Schizophrenia is a prevalent mental illness characterized by complex behavioral and emotional disturbances, with its underlying molecular mechanisms yet to be fully elucidated.

**Aim:**

This study aims to examine the neuroprotective effects of *Palmaria palmata* (*Palmaria p*.) on cognitive function in a schizophrenia mouse model.

**Methods:**

A total of 28 adult male SWR Swiss mice were used over a 30-day period. The animals were randomly divided into four groups (n = 7): control, cuprizone (CPZ) (0.2% CPZ in chow), CPZ + *Palmaria p.* (600 μg/kg bw/day via gavage), and *Palmaria p.* alone. The antioxidant activity of *Palmaria p.* was assessed using a radical scavenging assay. Behavioral assessments, hippocampal (HC) and frontal cortex (FC) gene expression analyses, and histopathological evaluations were conducted.

**Results:**

*Palmaria p.* demonstrated remarkable antioxidant activity against CPZ-induced oxidative stress. No notable effects were observed in spatial memory, the novel object recognition test (NORT), or anxiety-related behaviors. In the CPZ-treated group, Iba1 and CREB expression levels increased in both the hippocampus (HC) and frontal cortex (FC). In the CPZ + *Palmaria p.* group, Iba1 expression was reduced by approximately one-fold in the HC and two-fold in the FC, while CREB expression was decreased by approximately two-fold in both regions compared to the CPZ group, indicating attenuation of neuroinflammation and restoration of neuroplasticity. Immunohistochemical analysis revealed a notable decline in CD4^+^ expression following *Palmaria p.* administration, suggesting a decrease in the immunological response induced by CPZ.

**Conclusion:**

The results highlight the potential of *Palmaria p.* to enhance neuroplasticity and reduce neuronal inflammation associated with schizophrenia.

## 1 Introduction

Schizophrenia is a common and debilitating mental condition characterized by complex behavioral and emotional disturbances (Stępnicki et al., 2018). Although its exact pathophysiological mechanisms remain incompletely understood, substantial evidence implicates immunological dysregulation, neuroinflammation, and white matter abnormalities in its onset and progression (Orsolini et al., 2022). Studies have highlighted that impairments in spatial working memory (SWM) and anxiety symptoms are common in individuals with schizophrenia, negatively impacting their cognitive and functional outcomes (Wood et al., 2003; Bygrave et al., 2019). This disorder is also associated with structural and functional abnormalities in the hippocampus and frontal cortex, brain regions critical for memory, executive function, and decision-making (Wegrzyn et al., 2022; Weinberger, 1988; Mubarik and Tohid, 2016).

The cAMP-response element-binding (CREB) protein is a key regulator of neuroplasticity, synaptic strength, and cell survival; its dysregulation is a characteristic of numerous central nervous system disorders, including schizophrenia (Sawamura et al., 2008; Sakamoto et al., 2011). Furthermore, microglial activation, indicated by elevated expression of ionized calcium-binding adaptor molecule 1 (Iba1), contributes to neuroinflammation and synaptic dysfunction in schizophrenia (Drexhage et al., 2010; Prinz and Priller, 2014; Petrasch-Parwez et al., 2020). Although CD4^+^ T cells have been found in the central nervous system (CNS), their role in the healthy brain remains unclear. Notably, the absence of CD4^+^ T cells has been shown to disrupt microglial maturation, leading to immature neuronal connections and behavioral disorders, highlighting the critical interaction between the nervous and immune systems, especially during brain development (Pasciuto et al., 2020). However, existing data on microglial involvement in schizophrenia are inconsistent and, at times, contradictory, leaving key aspects of their role in the disease’s pathophysiology unresolved (Petrasch-Parwez et al., 2020; Hercher et al., 2014).

Current therapies, especially antipsychotics, target positive symptoms but have little effectiveness for cognitive deficiencies and are accompanied by side effects (Stroup and Gray, 2018). Thus, there is a rising interest in investigating safer, plant-based medicines to treat cognitive deficits and inflammation in schizophrenia (Lordan et al., 2011; Yousof et al., 2021). Marine algae, *Palmaria p.*, contain bioactive compounds with antioxidant and anti-inflammatory characteristics, making them potential candidates for neuroprotective therapy (Peñalver et al., 2020; El-Beltagi et al., 2022). *Palmaria palmata* includes vital amino acids, carotenoids, and sterols, which contribute to its health advantages, including the ability to improve cognitive function and reduce inflammation (Yuan et al., 2005; da Costa et al., 2021). Previous research has demonstrated *Palmaria palmata’s* therapeutic potential in illnesses such as multiple sclerosis and neurotoxicity, implying its significance to neurological disorders (Yousof et al., 2023). *Palmaria p.*, similar to most macroalgae, possesses a low lipid content (0.3–3.8% of dry weight); however, its lipids are highly accessible and abundant in eicosapentaenoic acid (EPA), an omega-3 polyunsaturated fatty acid (PUFA) recognized for its health advantages. EPA plays an essential role in the prevention of cardiovascular and neurological illnesses, demonstrating significant antioxidant and anti-inflammatory properties (da Costa et al., 2021; Yousof et al., 2023; Lopes et al., 2019). *Palmaria palmata* (dulse) extracts exhibited *in vitro* antioxidant and antiproliferative activities, largely due to their polyphenol content (Yuan et al., 2005).

Antipsychotic medications, referred to as neuroleptics or major tranquilizers, are utilized to manage schizophrenia, cerebral impairment, mania, toxic delirium, and various behavioral disorders. They are antagonists of dopamine receptors and possess limitations in efficacy and adverse effects. Contemporary drug design emphasizes the identification of compounds to alleviate negative symptoms, enhance cognitive deficits, and ensure improved tolerability for patients requiring long-term treatment, given the inadequate understanding of schizophrenia’s etiology (Stępnicki et al., 2018). Notwithstanding its promising phytochemical composition, a significant knowledge deficit exists concerning the potential application of *Palmaria p.* in neuropsychiatric disorders, including schizophrenia. In the meantime, no studies have systematically examined its efficacy—whether in crude or formulated forms—in addressing schizophrenia-related cognitive deficits, neuroinflammation, or critical molecular pathways such as CREB and Iba1. The mechanistic foundations of naturally derived therapies for restoring cognitive function are inadequately understood. This study seeks to fill this gap by assessing the neuroprotective effects of *Palmaria p.* in a cuprizone (CPZ)-induced murine model of schizophrenia, emphasizing behavioral outcomes and the modulation of CREB and Iba1 expression.

## 2 Materials and methods

### 2.1 Animals

A total of 28 adult male SWR Swiss mice, with approximate weights of 22 ± 2 g and ages of 8 ± 2 weeks. The mice were housed under ideal environmental conditions, including optimal temperature and humidity levels, a 12-h light/dark cycle, and unrestricted access to water and food. All experimental techniques adhered to the rules established by the Animal Care and Use Committee Office (ACUC) and received approval from the ACUC committee (ACUC-22-09-16).

### 2.2 Drug preparation

CPZ is a copper-chelating compound with a toxic effect on the nervous system. The administration of CPZ induces demyelinating lesions. The symptoms seen in this model are comparable to those seen in persons with schizophrenia (Herring and Konradi, 2011; Sun et al., 2021). CPZ (C9012-25G) was purchased from Thermo Fisher Scientific (Waltham, MA, USA). CPZ was freshly prepared daily and mixed with ground normal chow in 0.2% w/w of CPZ (Sun et al., 2021). *Palmaria p.* Leaf Powder Liquid Extract 2×4 oz. was purchased from HawaiPharm LLC (Hawaii, USA). The *Palmaria p.* solution was also freshly prepared each day, dissolved in distilled water, and administered via oral gavage at a volume of 0.2 mL per mouse, once daily (Yousof et al., 2023). The mice were weighed weekly to adjust the *Palmaria p.* dosage accordingly.

### 2.3 *Palmaria p.* extract diphenyl-1-picrylhydrazyl (DPPH) radical scavenging activity assay

The free radical DPPH is widely used to assess the antioxidant capacity of naturally occurring substances, as it serves as a simple, rapid, and effective method for assessing free-radical scavenging and hydrogen-donating abilities (Baliyan and Mukherjee, 2022).

The DPPH test is based on the removal of DPPH, a stabilized free radical. DPPH is a dark-colored crystalline substance consisting of stable free-radical particles. In solution, the DPPH radical appears dark purple; upon reduction to DPPH-H, it becomes colorless or pale yellow. Various plant extracts have demonstrated the ability to neutralize DPPH radicals *in vitro* (Baliyan and Mukherjee, 2022).

The 2,2-diphenyl-1-picryl-hydrazyl-hydrate (DPPH) free radical assay was conducted in accordance with the method of Raïnatou et al. (2016). Briefly, 100 μL of freshly prepared DPPH solution (0.1% in methanol) was added to 100 μL of the test sample in a 96-well plate (n = 6), and the mixture was incubated in the dark at room temperature for 30 min. After incubation, the decrease in DPPH color intensity was measured at 540 nm (Raïnatou et al., 2016).

The results were expressed as mean ± standard deviation (SD), and percentage inhibition was calculated using the following equation:


$$\text{Percentage inhibition}=\frac{(\begin{array}{l} \text{Average absorbance of blank}-\\ \text{average absorbance of test} \end{array})\;}{\left(\text{Average absorbance of blank}\right)}\times 100.$$

### 2.4 Experimental design

**Figure 1:**
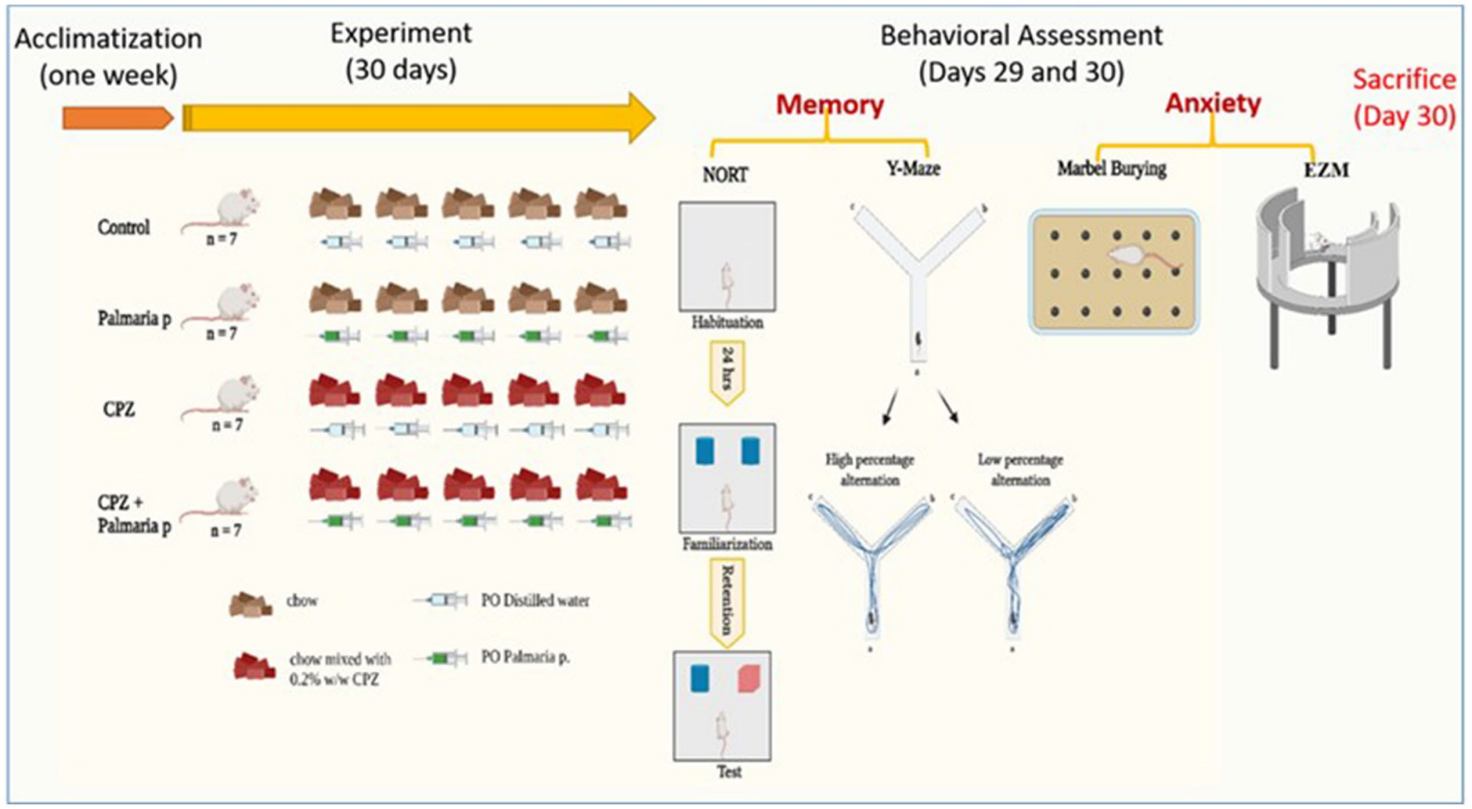
Timeline of the experiment with an overview of behavioral tasks. The presented graphical abstract illustrates the experimental methodology employed to examine the neuroprotective properties of *Palmaria palmata* in mitigating cognitive impairments and anxiety induced by CPZ in a mouse model of schizophrenia. The study included four distinct groups: control, *Palmaria p.* (*Palmaria palmata* treatment), CPZ (cuprizone treatment to cause cognitive deficits and anxiety), and CPZ *+ Palmaria p.* (co-treatment with *Palmaria palmata* to evaluate its protective benefits). After the 30-day experiment, behavioral evaluations were administered to assess the participants’ memory and anxiety levels. In this study, the NORT and Y-Maze tests were utilized to measure memory, whereas the marble burying test and elevated plus maze (EPM) were implemented to assess anxiety (n = 7/group).

Prior to the initiation of the experiment, the animals were housed for a period of 1 week to facilitate acclimatization. The mice were assigned randomly to four groups, with each group consisting of seven mice (n = 7). Control (fed with normal chow and 0.2 mL per oral distilled water daily for 30 days), CPZ (fed with CPZ-mixed chow 0.2% CPZ (w/w)) (Sun et al., 2021; Liu et al., 2017) and 0.2 mL of distilled water daily for 30 days for induction of schizophrenia model (Tian et al., 2018; Xu et al., 2010), CPZ + *Palmaria p*. (fed with CPZ-mixed chow and 0.2 mL of 600 μg/kg *Palmaria p.* daily for 30 days) (Yousof et al., 2023), and *Palmaria p.* only (fed with standard chow and 0.2 mL of 600 μg/kg *Palmaria p.* daily for 30 days). The dose of *Palmaria p.* was determined based on its neuroprotective effect, which was mentioned in previous literature (600 μg/kg bw/day by gavage) (Yousof et al., 2023) (Figure 1).

### 2.5 Behavioral tests

At the end of the experiment (Day 30), a battery of behavioral tests was carried out to assess cognitive function and anxiety-related behavior.

#### 2.5.1 Assessment of recognition memory

##### 2.5.1.1 Novel object recognition test (NORT)

The NORT is a behavioral assay of learning and memory, based on the assumption that mouse exhibit an unconditioned preference for exploring novel objects in a familiar environment (Moscardo et al., 2012; Wooden et al., 2021). Therefore, there is no need for reinforcement or multiple training sessions (Lueptow, 2017). NORT consists of 4 phases: habituation, familiarization, retention, and test. Habituation started 1 day before the familiarization phase, where the mouse is introduced to the arena (without objects) and the experiment environment to minimize anxiety and novelty confounding factors. Moreover, the habituation phase (15 min) helps increase the animal’s interest in the objects later in the subsequent phases (Carlini, 2011). This is followed by the familiarization phase (3 min), during which the mouse is introduced to two identical objects (called familiar objects) and allowed to explore them. Then the mouse is transferred to his home cage for the 10-min retention phase. This is followed by the test phase (3 min); the mouse is returned to the arena, and one previously explored object is replaced with a new (novel) object. If the mouse remembered the previous exposure to familiar objects, it would explore mainly the novel object.

On Day 1 (Day 29 of the experiment), mice underwent a habituation phase where each was placed individually in an open field arena for a duration of 15 min. On Day 2 (Day 30 of the experiment), during the familiarization phase, each mouse was exposed to two identical objects—labelled familiar object 1 (F1) and familiar object 2 (F2)—and allowed to explore them for 3 min. Following a 10-min interval, one of the familiar objects was replaced with a novel object, and the mouse was given an additional 3 min to explore this new setup (Figure 1). The EthoVision tracking system was used to measure both the time spent in proximity to each object and the frequency of nose-point interactions (sniffing) within each object zone (Alqurashi et al., 2022). The frequency of sniffing the objects and the discrimination index (DI) are calculated as follows:


$$\mathrm{DI}=\frac{\mathrm{TN}-\mathrm{TF}}{N+\mathrm{TF}},$$

in which *TN* is the time spent around the novel object and *TF* is the time spent around the familiar object (Alqurashi et al., 2022).

##### 2.5.1.2 Y-maze test

The Y-maze is a rapid and effective test used to assess short-term spatial memory in experimental animals. Structurally, it resembles a capital “Y,” consisting of three identical arms, converging at a 120ᴼ angle. Spatial working memory was assessed using the Y-maze through spontaneous alternation behavior. Each mouse was placed in one arm and allowed to freely explore all three for 5 min. Relying on the mice’s innate curiosity, they tend to prefer to enter previously unvisited arms (Kraeuter et al., 2019). Consecutive entries into three different arms without repetition are defined as spontaneous alteration behavior (Kraeuter et al., 2019; Hughes, 2004; Prieur and Jadavji, 2019). Re-entering a previously visited arm is considered indicative of memory impairment. An “arm entry” is recorded only when all four limbs of the mouse are within the arm (Sigurdsson and Duvarci, 2016; Shing et al., 2022).

In the current experiment, on day 29, the animals were moved from the animal house to the behavioral testing room and left to habituate for approximately 1 h. The apparatus comprised three identical arms, each measuring 60 cm in length and 10 cm in width, and labeled A, B, and C. Each mouse was introduced into one of the arms and allowed to explore the maze freely for a period of 5 min. Throughout the session, the order of arm entries was recorded. A *spontaneous alternation* was defined as consecutive entry into three different arms in sequence (e.g., ABC, CBA, or BAC) (Alqurashi et al., 2022). The total number of arm entries is counted to reflect mouse activity state among groups and calculate the percentage of spontaneous alteration. The percentage of alterations was calculated as follows:


$$\text{Percentage of alterations}=(\frac{\begin{array}{l} \text{Total Number}\;\\ \text{of alterations} \end{array}}{\begin{array}{l} \text{Total Number}\;\\ \text{ofArm Entries}-2 \end{array}})\times 100,$$

in which alteration in a consecutive entry into three different arms: ABC, CBA, BCA, and BAC.

#### 2.5.2 Assessment of anxiety-related behaviors

##### 2.5.2.1 Elevated zero maze (EZM)

**Figure 2:**
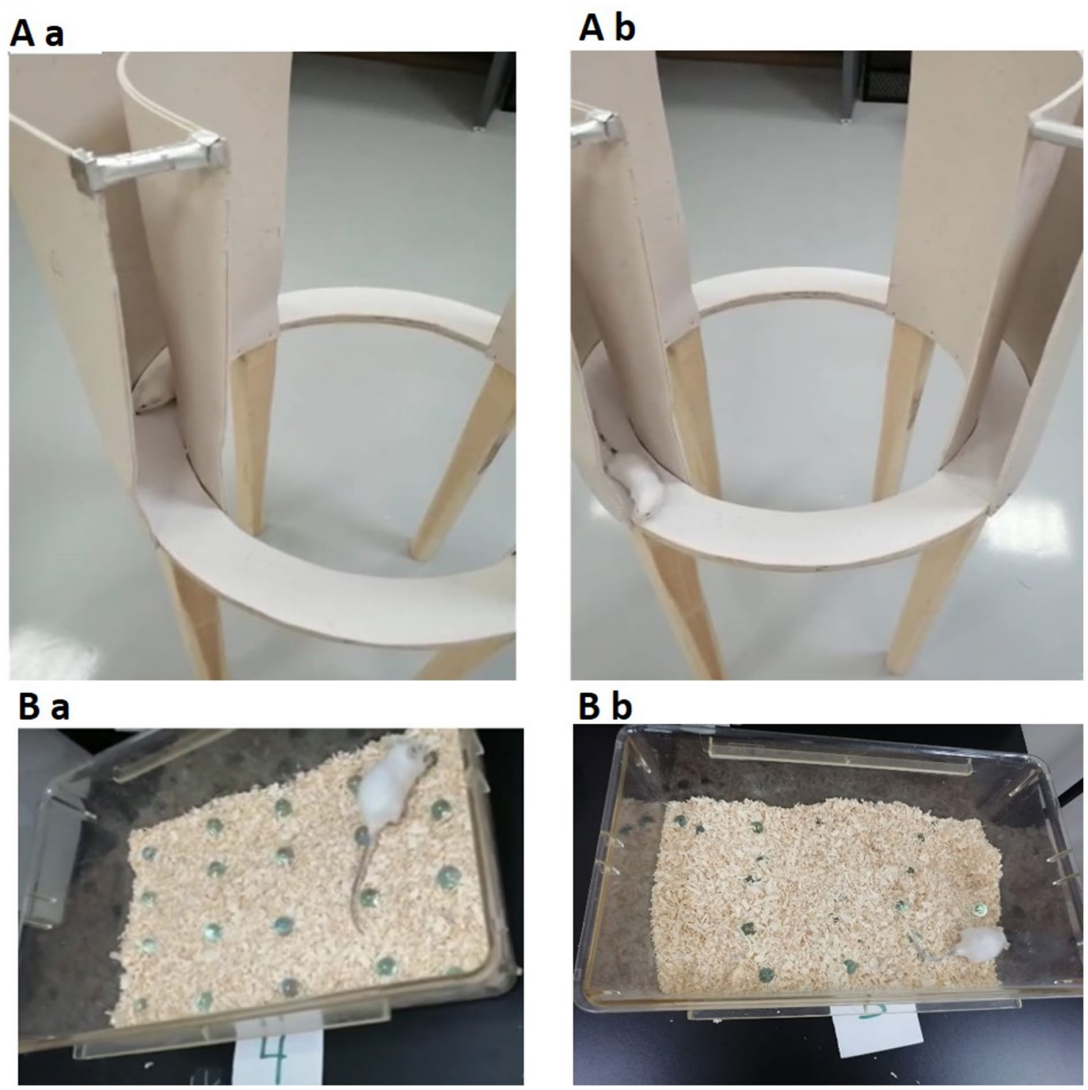
The upper figure **(Aa,Ab)** demonstrates the EZM. The lower part of the figure **(Ba,Bb)** demonstrates the MBT. Both tests are used for assessing anxiety-related behavior in mice. EZM, elevated zero maze; MBT, marble burying test (n = 7/group).

The zero maze test, which is based on mice’s inherent aversion to elevated and open mazes, is a pharmacologically validated anxiety measure in animal models (Tucker and McCabe, 2021). The equipment is raised 50 cm from the ground. The EZM is a 60-cm-diameter annular beige platform divided into four equal quadrants. The remaining two “closed” quadrants were bordered by opaque walls, while the two opposite quadrants were “open.” The lane width in the quadrants was 5 cm. Overhead fluorescent bulbs supplied illumination to the maze (Tucker and McCabe, 2021). Each mouse was placed in a closed section of the zero maze at the beginning of the assignment and given 10 min to explore the maze. The head stage location of the animals was carefully noted. The closed segment time (the percentage of time a mouse spent in the closed segment during a 10-min session) and closed segment entries for each mouse were then determined (the number of times that a mouse moved from the open segment into the closed segment of the maze during the 10-min session), Figures 2Aa,Ab. The maze was cleaned with 70% ethanol between trials (Braun et al., 2011).

In the present experiment, on day 29, the animals were moved from the animal house to the behavioral testing room and left to habituate for about 1 h. Each mouse was placed in the center of the apparatus, and its behavior was monitored for a duration of 5 min. Anxiety levels were quantified using the anxiety index, calculated with the following formula:


$$\text{Anxiety index}=1-(\begin{array}{l} {}[\text{open}\;\mathrm{arm}\;\text{time}/5\;\min]+\\ \left[\text{open}\;\mathrm{arm}\;\text{entry}/\text{total entry}\right] \end{array})/2.$$

##### 2.5.2.2 Marble burying test (MBT)

Mice engage in a variety of species-specific behaviors, including digging and burrowing. In the laboratory, mice dig vigorously in deep bedding, such as wood chips. Drugs and strain differences affect this behavior (Angoa-Pérez et al., 2013). In the current experiment, on day 30, the animals were moved from the animal house to the behavioral testing room and left to habituate for about 1 h. A cage (17.5 × 10 × 5.5 inches) was filled to a depth of about 5 cm with husk bedding material evenly dispersed across the entire cage on a flat surface. The 20 glass marbles (1.4 cm in diameter) were then equally spaced on the bedding’s surface in a 4 × 5 grid. During the testing phase, each mouse was placed in a cage and given 30 min to investigate (Figures 2Ba,Bb). The mice were removed from the cage at the end of the experiment, and the number of marbles buried up to 2/3 of their depth in bedding was counted (Angoa-Pérez et al., 2013; Lazic, 2015).

#### 2.5.3 Animal sacrifice

Following behavioral assessments (on day 30), mice were anesthetized using isoflurane and subsequently euthanized by decapitation in accordance with the guidelines of the King Fahd Medical Research Center (Labban et al., 2021). The entire brain was promptly extracted and rinsed with saline. The hippocampus and prefrontal cortex were then dissected from each brain, and the *left hippocampus* was preserved in *RNAlater solution* (Invitrogen) for subsequent gene expression analysis.

#### 2.5.4 RNA extraction and cDNA synthesis

The total RNA was extracted from the frontal cortex using a TRIzol® Reagent purchased from Sigma India. In short, after being taken out of the RNA-later solution at −80°C, the brains were left to thaw at ambient temperature. In tubes, 50–60 mg of homogenized hippocampus/frontal cortex tissue was used for RNA extraction, following the manufacturer’s instructions. A NanoDrop spectrophotometer was used to measure the concentration and purity of the RNA, and the samples were kept at −80°C. The RNAs were reverse transcribed to cDNA using a Maxima First Strand cDNA Synthesis Kit K1641 (Thermo-Fisher Scientific) following the manufacturer’s instructions.

#### 2.5.5 Gene expression analysis

After the animal had been sacrificed, the brain was removed and divided into two parts: one for the RNA later and the second part was frozen for histological analysis. This study examined the amounts of mouse CREB and Iba genes mRNA expression in the hippocampus and frontal cortex regions. The primers were designed using the NCBI primer design tool. In brief, 300 nM forward and reverse primers were combined with PowerUp SYBR Green PCR Master Mix (Applied Biosystems) and 10 ng of the generated cDNA. The PCR process was initiated according to the kit’s instructions: (1) UDG activation for 2 min at 50°C (one cycle); (2) Dual-LockTM DNA polymerase activation for 2 min at 95°C (one cycle); and (3) forty cycles of denaturation for 15 s at 95°C followed by annealing/extension for 1 min at 60°C (if primer Tm ≥ 60°C). The annealing temperature (52–59°C) was established for each primer set, including primers with a Tm less than 60°C, and the temperature was eventually raised to 72°C. For the purposes of this investigation, the housekeeping gene was GAPDH. The StepOne device (Applied Biosystems) was used to conduct the research. The relative fold change in gene expression was determined using the delta–delta Ct technique.

#### 2.5.6 Histological staining

##### 2.5.6.1 H & E

The study involved adult mice under standard laboratory conditions, euthanized after experimental treatments, and brains removed and placed in cold phosphate-buffered saline. The hippocampus and frontal cortex were dissected and fixed in formalin for 24–48 h. The specimens were preserved in 10% neutral formalin for a duration of 10 days.

They underwent dehydration in increasing concentrations of ethyl alcohol (50, 70, 96%, and absolute alcohol). The specimens were cleared in xylene. The tissues were subsequently infused with soft paraffin by subjecting them to multiple baths of melted wax (melting point 50°C) in an oven. Ultimately, embedding in hard paraffin was accomplished by immersing the tissues in molten wax (melting point 55°C), which was then poured into a mold and subsequently cooled to create paraffin blocks encasing the tissues. Serial sections measuring 5–7 micrometers in thickness were prepared, affixed to slides using an adhesive, and subsequently stained with hematoxylin and eosin (Bancroft and Gamble, 2013). All sections are analyzed using an electron microscope, and images are captured with a digital pathology scanner at Alborg Pathology Lab.^1^ Some of these sections were analyzed using the Olympus light microscope and photographed with the Olympus E330 microscope. Magnifications were noted in the sections examined in the Histopathological Laboratory at the Faculty of Medicine in Rabigh, King Abdulaziz University.

#### 2.5.7 Immunohistochemistry antibody against CD4

The study involved cryoprotecting fixed tissues in 30% sucrose in PBS, embedding them in OCT compound, and freezing them at −20°C. Coronal hippocampus and frontal cortex sections were cut and mounted onto glass slides coated with poly-L-lysine. The sections were stored at −80°C until immunostaining. The sections were blocked with 5% normal goat serum and incubated with a primary antibody against CD4 overnight at 4°C. After washing three times in PBS, they were incubated with a fluorophore-conjugated secondary antibody for 1 h in the dark. The sections were counterstained with DAPI to visualize nuclei and mounted with an anti-fade mounting medium. The sections were examined under a fluorescence microscope using appropriate filters, and images were captured using a digital camera attached to the microscope. Image analysis was performed using software to quantify CD4-positive cells in the hippocampus.

### 2.6 Data analysis

Data were analyzed and represented using Microsoft Excel, GraphPad Prism, and IBM SPSS software (version 23). We tested normality using the Shapiro–Wilk test. We found that the behavioral test results are not normally distributed in all groups. Therefore, we analyzed the Kruskal-Wallis test to assess the significant differences (*p* ≥ 0.05 → no significant difference between the groups; retain null hypothesis). The normally distributed data were expressed in means and standard deviations, and the means were compared using one-way or two-way ANOVA. If the ANOVA was significant, the post-hoc LSD test was employed to determine group differences. The artwork was created using Mind the Graph, BioRender, and Microsoft PowerPoint.

## 3 Results

### 3.1 The DPPH (2,2-diphenyl-1-picrylhydrazyl) assay

The DPPH assay revealed that the sample exhibited an antioxidant capacity of 0.720 ± μM T eq/mg. This finding suggests that the sample has a measurable ability to neutralize free radicals, thereby exhibiting significant antioxidant potential.

### 3.2 Behavioral analysis

#### 3.2.1 Episodic memory assessment via NORT

**Figure 3:**
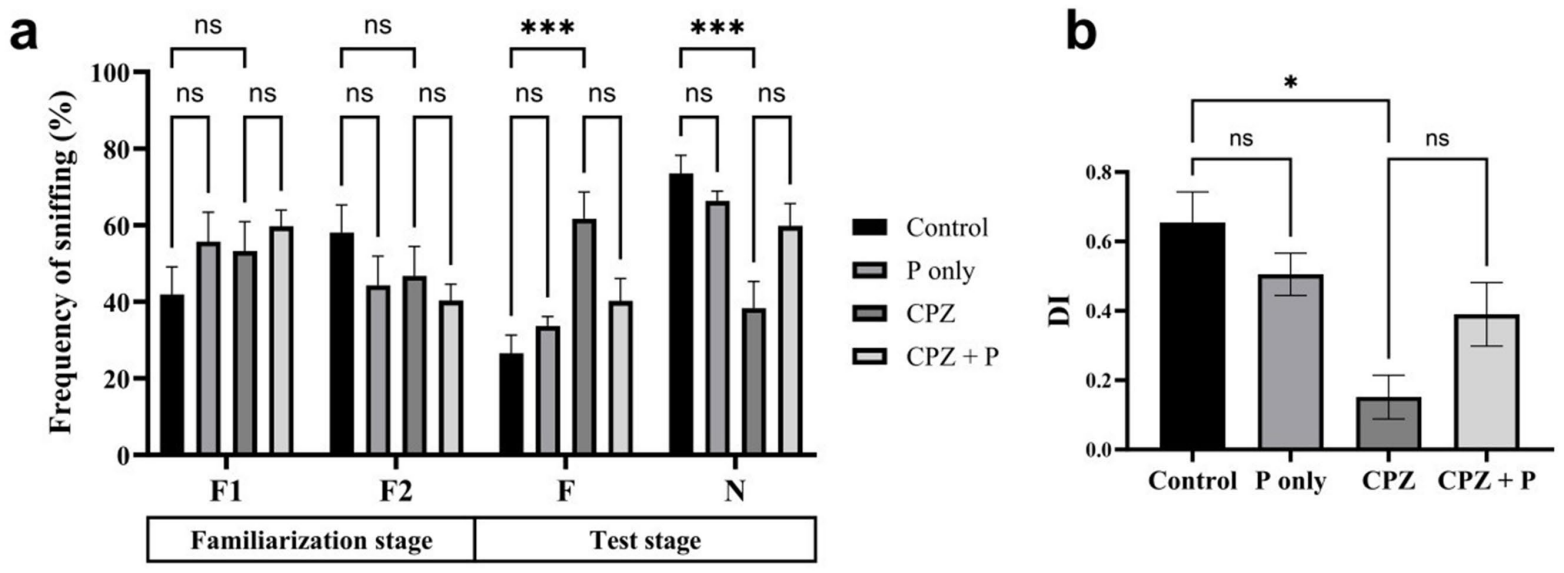
CPZ treatment impaired social recognition memory, as shown by the lack of preference for the novel mouse during the test stage **(a)** and the significantly reduced discrimination index (DI) **(b)**. Control and P-only groups exhibited normal social memory, demonstrated by a preference for the novel over the familiar mouse and higher DI scores. Co-treatment with P (CPZ + P) showed a non-significant trend toward improvement, indicating that P may offer partial protection against CPZ-induced deficits (n = 7/group).

In the NORT, no significant differences were observed among groups during the familiarization stage (F1 and F2) (Figure 3a). During the test stage, both the control and *Palmaria-*only groups exhibited a significant preference for the novel object over the familiar one, as reflected by increased sniffing frequency, suggesting intact episodic memory. However, CPZ-treated mice, with or without *Palmaria* co-treatment, failed to show this preference, indicating impaired episodic memory. To assess the effects of treatment and stimulus type (familiar vs. novel) on NOR, we applied a two-way ANOVA. This analysis revealed a significant interaction between treatment and stimulus in the test stage, justifying further *post hoc* comparisons. The discrimination index (DI) was significantly reduced in the CPZ group compared to controls (Figure 3b). While the *CPZ+P* group demonstrated a trend toward improved DI values compared to CPZ alone, this did not reach statistical significance.

#### 3.2.2 Spatial memory assessment via Y-maze

**Figure 4:**
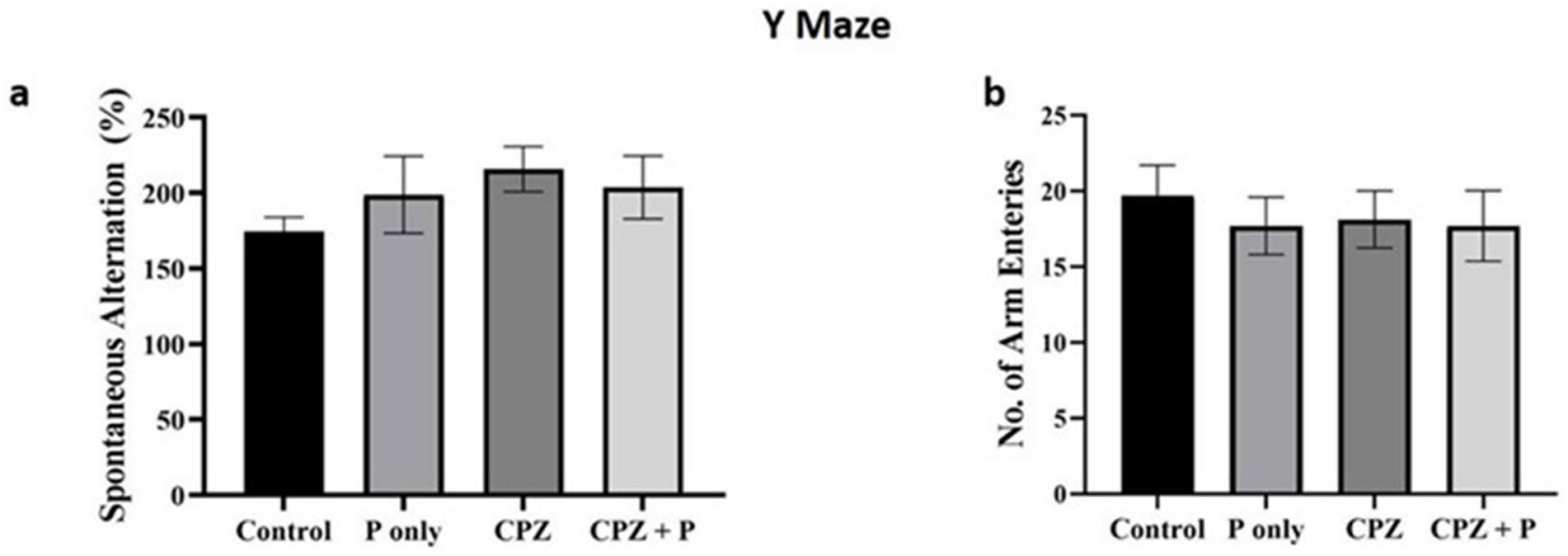
Showing the anxiety-related tests. **(a)** The number of spontaneous alterations (%) and the number of entries were assessed using the Y maze. **(b)** The number of closed arm entries and the time elapsed in the closed arm were compared using the elevated zero maze. The number of buried marbles was assessed via the marble burying test. *p* > 0.05 in the three tests. CPZ = cuprizone, P = *Palmaria p.* (n = 7/group).

All the treated groups revealed insignificant enhancement of the spontaneous alterations and insignificant decrease in the arm entries (*p* > 0.05, Figure 4). Collectively, the assessment of spatial memory by the Y-maze revealed no statistically significant changes among all the treated groups. The percentage of spontaneous alterations and the number of entries are often employed to assess spatial working memory and exploratory activity, respectively. The absence of statistically significant differences indicates that the experimental interventions did not substantially affect the animals’ cognitive abilities or exploratory conduct.

#### 3.2.3 EZM and MBT for anxiety-related behavior assessment

**Figure 5:**
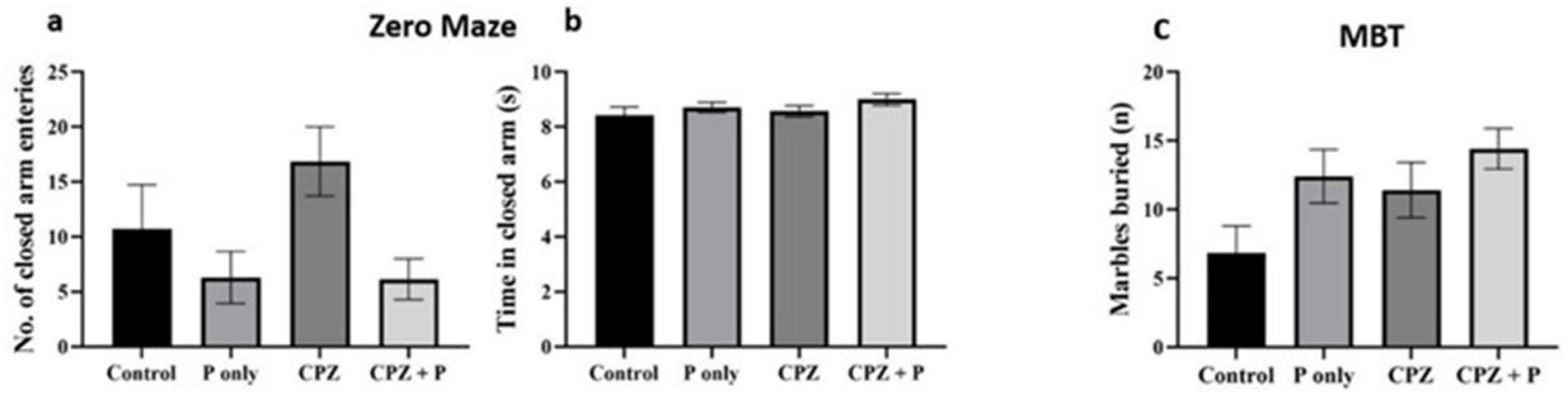
Assessment of the anxiety-related behavior via the elevated zero mazes **(a,b)** and the marble burying test **(c)**. The analysis of the number of closed arm entries and time spent in closed arms did not yield statistically significant differences between the groups (*p* > 0.05), suggesting a negligible effect on anxiety-related behavior. The number of buried marbles did not exhibit statistically significant changes across the groups (*p* > 0.05), implying an absence of a major impact of *Palmaria palmata* on anxiety-related compulsive behavior. CPZ = cuprizone, P = *Palmaria p.*

The elevated zero maze and marble burying tests were used to evaluate how treatments affected anxiety-related behavior. Neither the quantity of entries in the elevated zero maze’s closed arms (*p* > 0.05) nor the amount of time spent there (p > 0.05) varied substantially across groups (Kruskal–Wallis, *p* > 0.05) (Figures 5a,b). Similarly, the number of buried marbles in the MBT was similar between groups (*p* > 0.05). Together, these findings show that neither CPZ, palmaria extract, nor their combination significantly altered anxiety-related behavior in the investigated settings (Figure 5c).

### 3.3 Gene expression results

*Palmaria p.* was found to have the potential to enhance the CREB and Iba1 expression in both the hippocampus and frontal cortex.

#### 3.3.1 Iba1 and CREB expression in the hippocampus (HC) and frontal cortex (FC)

The effects of *Palmaria p.*, CPZ, and their combination on Iba1 and CREB expression in both the HC and frontal cortex FC were evaluated (Table 1). These markers are indicative of microglial activation and neuroplasticity, respectively, and provide insight into region-specific responses to treatment. In the control group, the expression levels of both Iba1 and CREB in the HC and FC were set to 1, and the groups were compared as fold changes.

**Table 1:** Results of gene expression.

Gene expression region/Group	Iba1-HC	Iba1-FC	CREB-HC	CREB-FC
	Mean ± SEM	Mean ± SEM	Mean ± SEM	Mean ± SEM
Control	1 ± 0.001	1 ± 0.001	1 ± 0.001	1 ± 0.001
*Palmaria p.*	0.25 ± 0.11	0.47 ± 0.10	0.2863 ± 0.09	0.85 ± 0.25
CPZ	3.67 ± 0.54^ab^	3.34 ± 0.78^ab^	3.55 ± 0.97 ^ab^	2.85 ± 0.48^ab^
CPZ *+ Palmaria p.*	2.40 ± 0.45^b^	1.60 ± 0.27^c^	1.15 ± 0.26^c^	1.61 ± 0.33

CPZ = cuprizone, P = *Palmaria p.*, a = compared to control group, b = compared to *Palmaria palamta* group, c = compared to CZP group, *p* < 0.05.

In the CPZ-treated group, both brain regions showed a marked increase in Iba1 expression. In the hippocampus and frontal cortex, Iba1 levels were about 2.5-fold higher (*p* < 0.05) compared to the control and *Palmaria p.* groups, reflecting significant microglial activation and neuroinflammation. CREB expression was also significantly elevated, about 2.5-fold in the hippocampus and 2-fold in the frontal cortex (*p* < 0.05), which might indicate a compensatory response to CPZ-induced damage.

However, in the combination group (CPZ + *Palmaria p.*), there was a significant reduction in Iba1 expression in both the hippocampus (about 1-fold reduction) and frontal cortex (about 2-fold reduction) compared to CPZ alone (*p* < 0.05). This suggests that *Palmaria p.* mitigates CPZ-induced neuroinflammation in both regions, reducing Iba1 expression more pronounced in the frontal cortex. Similarly, CREB expression in the combination treatment group was significantly lower than in the CPZ-only group, showing approximately a 2-fold reduction in both the frontal cortex and hippocampus (*p* < 0.05) compared to CPZ alone. This might indicate that *Palmaria p.* modulates CREB expression, restoring it toward control levels and possibly offering protection against CPZ-induced disruptions in neuroplasticity.

Collectively, *Palmaria p.* appears to have potential protective effects against CPZ-induced neuroinflammation and dysregulation of neuroplasticity-related pathways in both the hippocampus and frontal cortex. The combined treatment with CPZ and *Palmaria p*. reduced the over-expression of genes observed with CPZ alone treatment in terms of microglial activation (Iba1) and CREB expression in both brain regions.

### 3.4 Histopathology and immuno-histochemistry

This study reveals the histopathological findings in a model of CPZ-induced schizophrenia, a control group, and a *Palmaria p.-*treated group. The examination focused on the hippocampus and the prefrontal cortex. Tissue samples from these regions were stained using H&E and immunohistochemistry techniques and then observed at various magnifications.

#### 3.4.1 Hematoxylin and eosin staining of the hippocampus and frontal cortex

**Figure 6:**
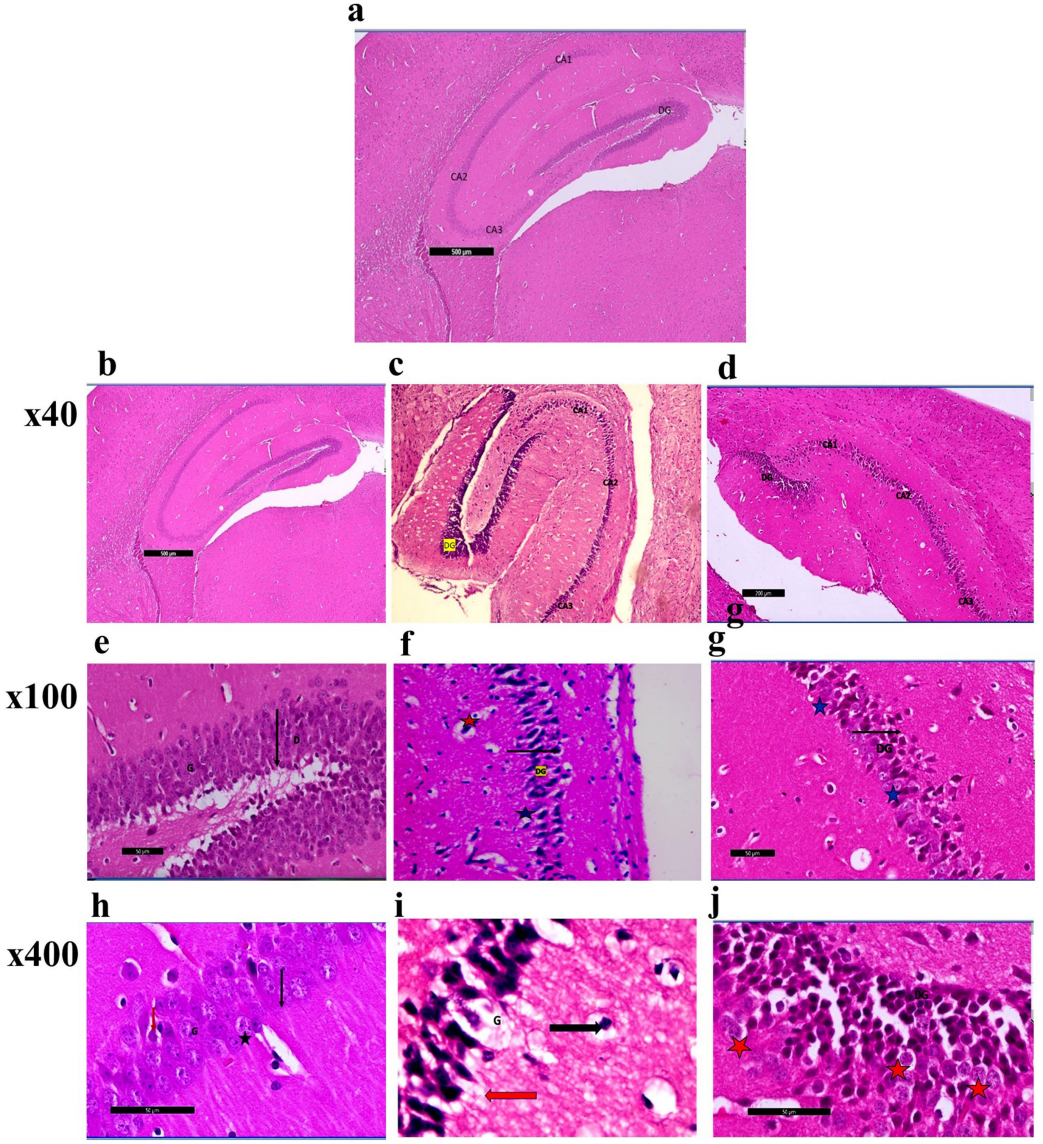
**(a)** A photomicrograph of sections (control –ve group) of adult male SWR Swiss mice showed the normal architecture of the different parts of the hippocampus: CA1, CA2, CA3, and dentate gyrus (DG), H&E; ×40. **(b)** A photomicrograph of sections (control +ve group) of adult male SWR Swiss mice showed the normal architecture of the different parts of the hippocampus: CA1, CA2, CA3, and dentate gyrus (DG), H&E; ×40. **(c)** A photomicrograph of Section (affected group) of adult male SWR Swiss mice showed the normal architecture of the different parts of the hippocampus: CA1, CA2, and CA3, with apparently increased thickness in the marked area of the dentate gyrus (DG), H&E; ×40. **(d)** A photomicrograph of sections (treated group) of adult male SWR Swiss mice showed the hippocampus, showing the different parts of the hippocampus: CA1, CA2, and CA3. Note the apparent decrease in the thickness of the dentate gyrus (G), H&E; ×40. **(e)** A photomicrograph of sections (control +ve group) of adult male SWR Swiss mice showed the normal architecture of the dentate region (black arrow D), formed mainly of an aggregation of granular cells (G), H&E; ×100. **(f)** A photomicrograph of sections (affected group) of adult male SWR Swiss mice showed higher magnification of the dentate gyrus (black arrow DG) with marked loss of its normal architecture (loss of granular layer) and marked thickness with apparently packed cells; note the shrunken cells (blue star) as well as the ruptured one (red star), H&E; ×100. **(g)** A photomicrograph of sections (treated group) of adult male SWR Swiss mice showed higher magnification of the dentate gyrus (black arrow DG), with a regain of its thickness with apparently moderate appearance of the granular cells (blue star), H&E; ×100. **(h)** A photomicrograph of sections (control +ve group) of adult male SWR Swiss mice showed the normal architecture of the dentate gyrus, formed mainly of an aggregation of granular cells (G) that appear rounded in shape with vesicular nuclei and prominent nucleoli (black star). Note the apparent presence of a pyramidal cell (red arrow), H&E; ×400. **(i)**: A photomicrograph of sections (affected group) of adult male SWR Swiss mice showed higher magnification of the dentate gyrus. Note the complete loss of granular cells (G). Pyramidal cells showed open-face nuclei and prominent nucleoli (black arrow). Other cells appear markedly dark, shrunken, and detached from the surrounding (red arrow), H&E; ×400. **(j)** A photomicrograph of sections (treated group) of adult male SWR Swiss mice showed higher magnification of the dentate gyrus (DG). Note marked regain of granular cell layer (G). Other cells appear markedly dark, shrunken, and detached from the surrounding (red star), H&E; ×400.

Photomicrographs obtained from adult male SWR Swiss mice in the control groups demonstrated the typical anatomical integrity of various regions inside the hippocampus, namely CA1, CA2, CA3, and the dentate gyrus (DG) (Figures 6a,b,e). Higher magnification revealed the clustering of granular cells characterized by their rounded morphology, vesicular nuclei, and prominent nucleoli. Additionally, pyramidal cells were observed (Figures 6c,h). In the CPZ group (Figures 6c,f,i). Photomicrographs revealed the presence of the typical hippocampal architecture, encompassing CA1, CA2, and CA3. However, there was an observable augmentation in the thickness of the dentate gyrus region.

Nevertheless, a notable loss of the typical dentate gyrus structure, including the granular layer, was demonstrated when observed at greater magnifications. The cells had a dense and shrunken morphology, with some showing signs of rupture. A complete loss of granular cells was seen, but pyramidal cells exhibited open-face nuclei and prominent nucleoli. The remaining cells had a dark appearance, reduced size, and detachment from the adjacent tissue environment. Photomicrographs in the CPZ + *Palmaria p.* (Figure 6d) group revealed the presence of the hippocampus, namely the CA1, CA2, and CA3 areas. The dentate gyrus exhibited a progressive reduction in thickness. A moderate preservation of the granular cells within the dentate gyrus was observed at higher magnification. Nevertheless, the remaining cells had a distinct dark appearance, diminished size, and detachment from the adjacent tissue (Figures 6g,j).

**Figure 7:**
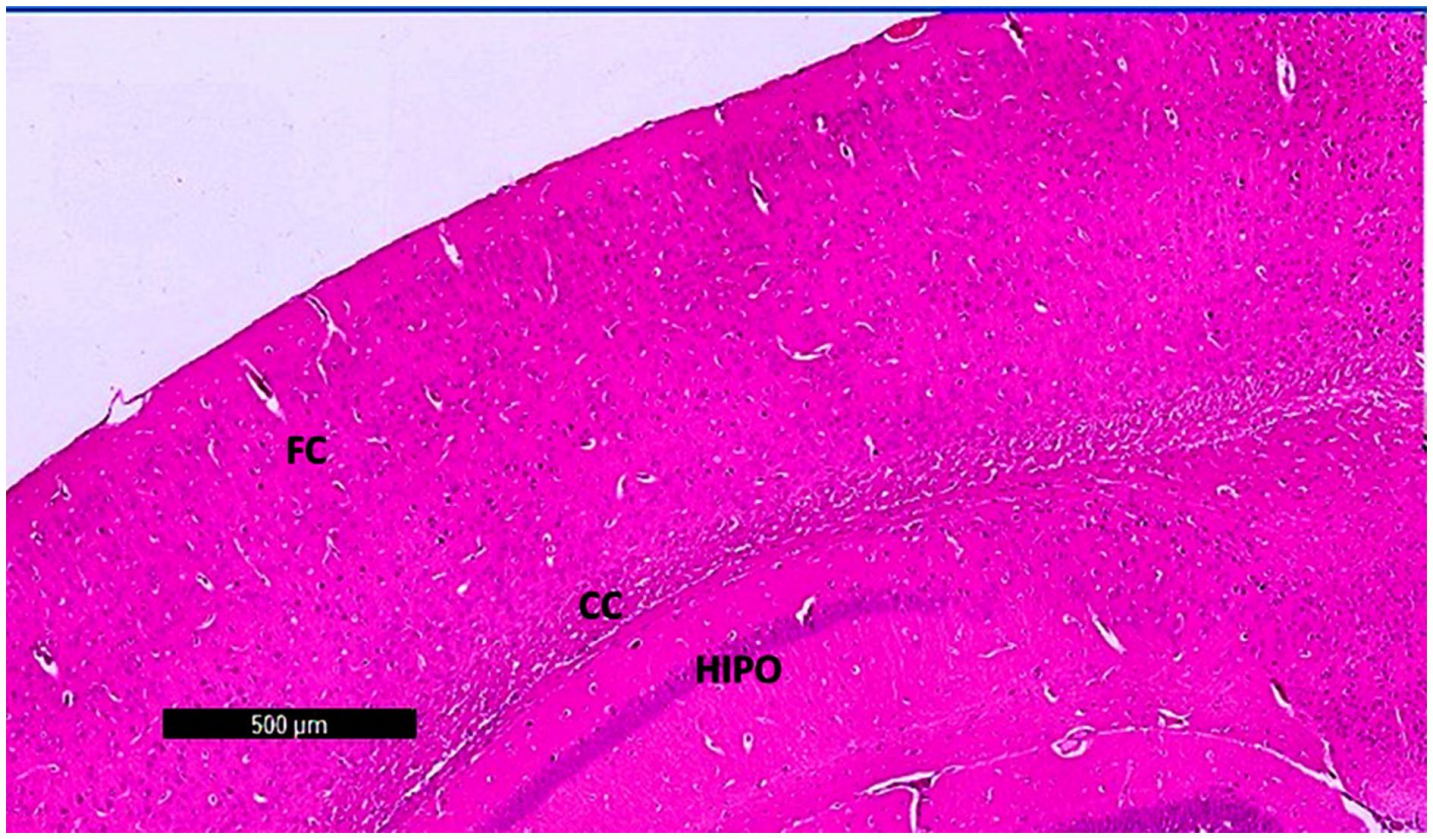
A photomicrograph of sections (control –ve group) of adult male SWR Swiss mice showed the normal architecture of the different parts of the brain, showing frontal cortex (FC), corpus callosum (CC), and hippocampus (Hipo). H&E ×40.

**Figure 8:**
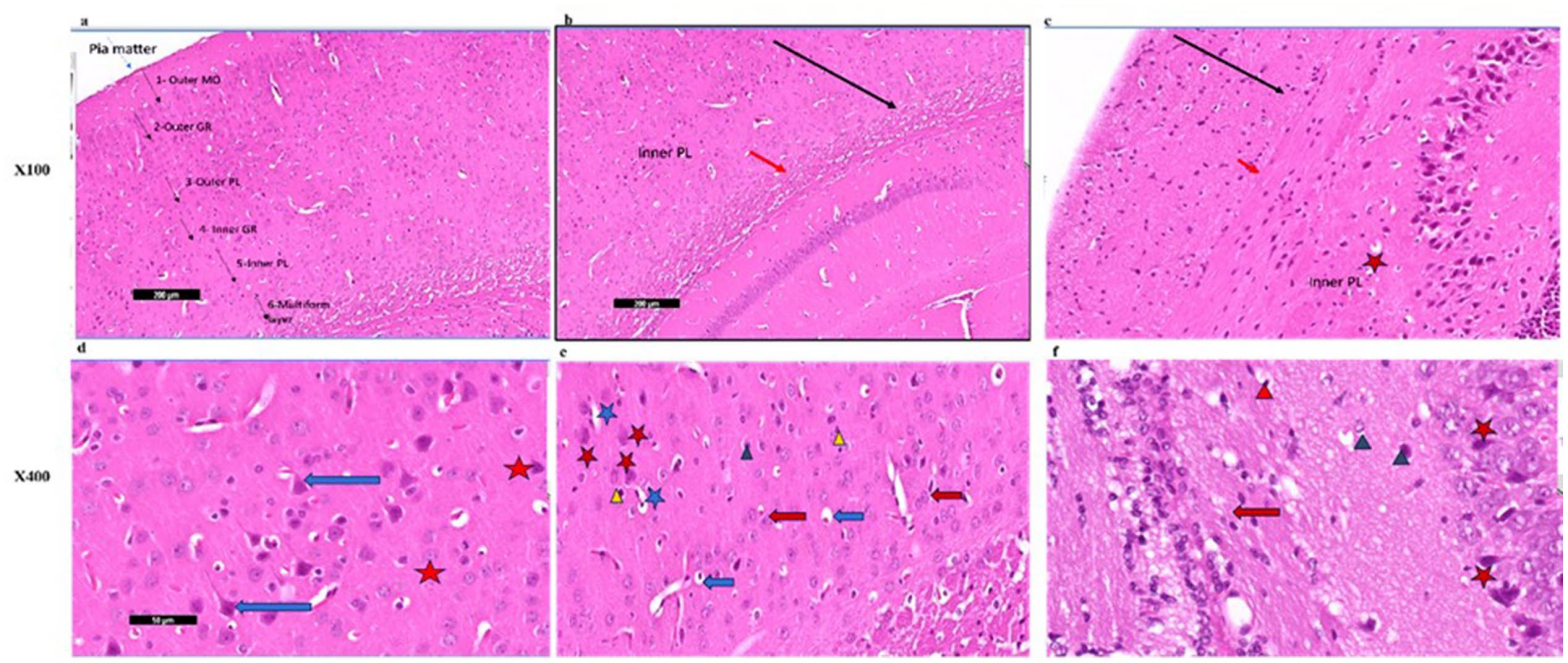
**(a)** A photomicrograph of the *Palmaria p.* (control +ve) group of adult male SWR Swiss mice showed the normal architecture of the frontal cortex covered with pia matter and its six different layers (1—outer molecular layer, 2—outer granular layer, 3—outer pyramidal cell layer, 4—inner granular layer, 5—inner pyramidal cell layer, and 6—multiform layer), H&E ×100. **(b)** A photomicrograph of CPZ (affected group) of adult male SWR Swiss mice showing apparently amalgamated layers (black arrow) of frontal cortex and thickening of the multiform layer (red arrow). Apparently normal appearance of inner pyramidal cell layer, H&E ×100. **(c)** A photomicrograph of CPZ + *Palmaria p.* (treated group) of adult male SWR Swiss mice showing apparently amalgamated layers (black arrow) of frontal cortex, a mild decrease in the thickening of the multiform layer (red arrow), and a mild decrease in vacuolations. Note the open window cells of the pyramidal cell layers (red star), H&E ×100. **(d)** A photomicrograph of *Palmaria p.* (control +ve group) of adult male SWR Swiss mice showed the normal architecture of large pyramidal neurons with apical dendrites (blue arrow). Note the multiform layer of the frontal cortex shows pleomorphic nuclei of different cells (pyramidal and granular cells, red star, H&E; ×400). **(e)** A photomicrograph of the CPZ group shows a loss of shape in the pyramidal cell layer, with loss of its dendrites (red star) as well as ruptured cells with detached cytoplasm (blue star). Inner granular cell layer showing packed cytoplasmic cell layer (green triangle) as well as pyknotic nuclei (yellow triangle). Multiform cell layer showing multinucleated cells. Note vacuolated cells (blue arrow), apparently cells with eccentric nuclei, H&E ×400. **(f)** A photomicrograph of the *CPZ+ Palmaria p.* group in adult male SWR Swiss mice showing a normal pyramidal cell layer with regrowth of its dendrites (red star). The inner granular cell layer shows a normal pleomorphic cell layer (green triangle), but the appearance of pyknotic nuclei (red triangle) is still notable. Multiform cell layer showing multinucleated cells (red arrow). Note a decrease in the vacuolated cells, H&E ×400 (n = 7/group).

Photomicrographs of frontal cortex sections from adult male SWR Swiss mice in the control group revealed normal architecture across its six layers, with large pyramidal neurons and a diverse array of cell types in the multiform layer (Figures 7, 8a,d). In the CPZ group, there was a notable decrease in frontal cortex thickness, amalgamation of layers, and thickening of the multiform layer (Figure 8b). Higher magnification showed loss of shape and dendrites in pyramidal cells, with some cells appearing ruptured and cytoplasm detached. The inner granular cell layer exhibited packed cytoplasmic cells with pyknotic nuclei, and the multiform cell layer displayed multinucleated and vacuolated cells with eccentric nuclei (Figure 8e). In contrast, the CPZ + *Palmaria p.* group showed amalgamated layers with a mild reduction in multiform layer thickening and fewer vacuolations. Pyramidal cells in the treated group regained their normal shape and dendrites, and the inner granular cell layer exhibited normal pleomorphic cells, though pyknotic nuclei persisted. The multiform cell layer showed a decrease in vacuolated cells and maintained multinucleated cells (Figures 8c,f).

#### 3.4.2 Immunohistochemistry staining of the hippocampus and frontal cortex

**Figure 9:**
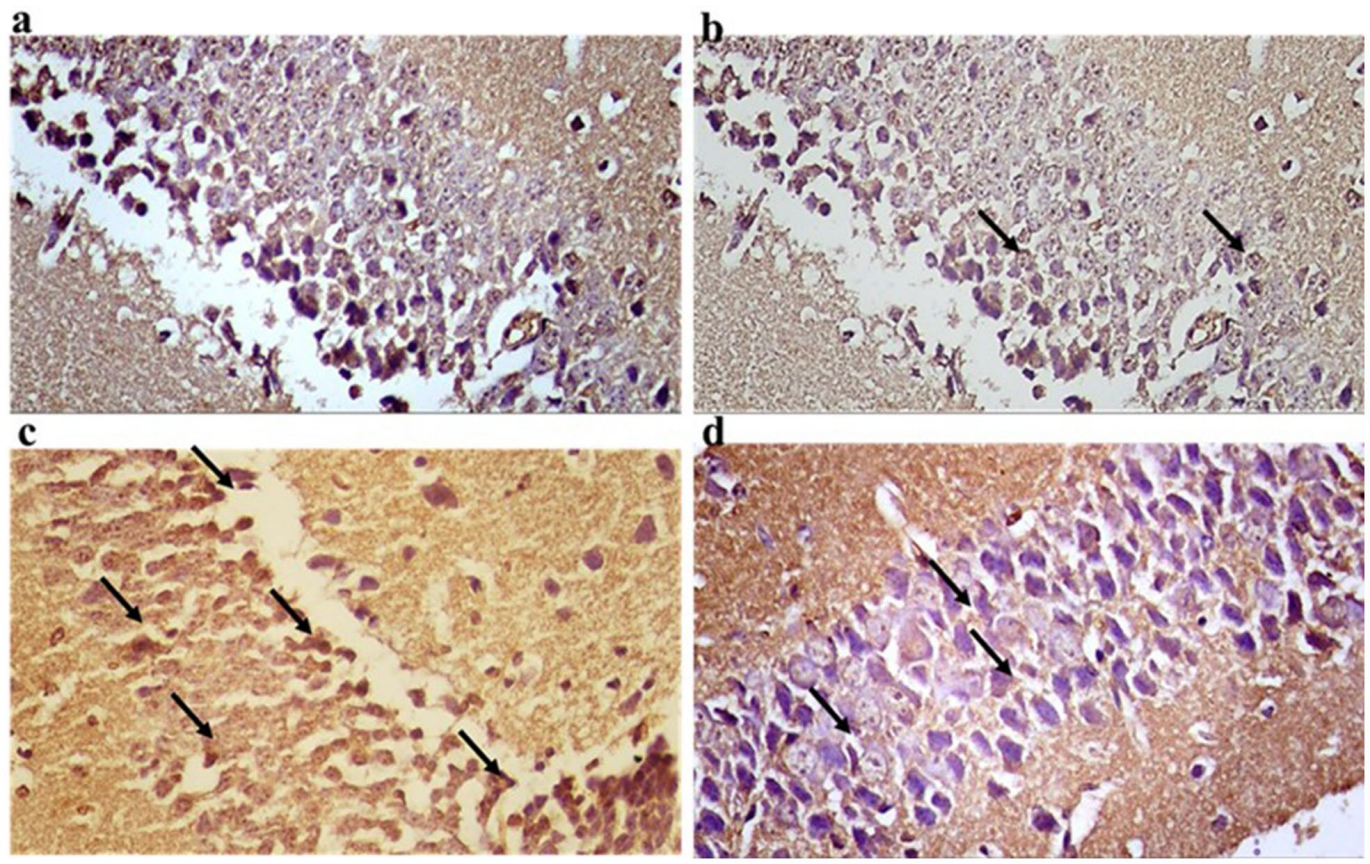
**(a)** A photomicrograph of the control –ve group of adult male SWR Swiss mice showed the normal architecture of the dentate gyrus of the hippocampus (DG) exhibiting CD4 mild immunoreaction of the nerve fibers, CD4 ×40. **(b)** A photomicrograph of the *Palmaria p.* only group of adult male SWR Swiss mice showed the normal architecture of the dentate gyrus of the hippocampus (DG) exhibiting CD4 Mild immunoreaction of the nerve fibers, CD4 ×40**. (c)** A photomicrograph of the CPZ group showed the normal architecture of the dentate gyrus of the hippocampus (DG) exhibiting CD4-marked immunoreaction of the nerve fibers, CD4 ×40. **(d)** A photomicrograph of CPZ + *Palmaria p.* showed the normal architecture of the dentate gyrus of the hippocampus (DG) exhibiting CD4 moderate immunoreaction of the nerve fibers, CD4 ×40 (n = 7/group).

Photomicrographs in the control groups showed the normal architecture of the dentate gyrus of the hippocampus (Figures 9a,b). The sections exhibited mild CD4 immunoreactivity in the nerve fibers. Photomicrographs from the CPZ group showed the normal architecture of the dentate gyrus of the hippocampus. The sections exhibited marked CD4 immunoreactivity in the nerve fibers (Figure 9c). Photomicrographs from the CPZ + *Palmaria p.* group showed the normal architecture of the dentate gyrus of the hippocampus. The sections exhibited moderate CD4 immunoreactivity in the nerve fibers (Figure 9d).

**Figure 10:**
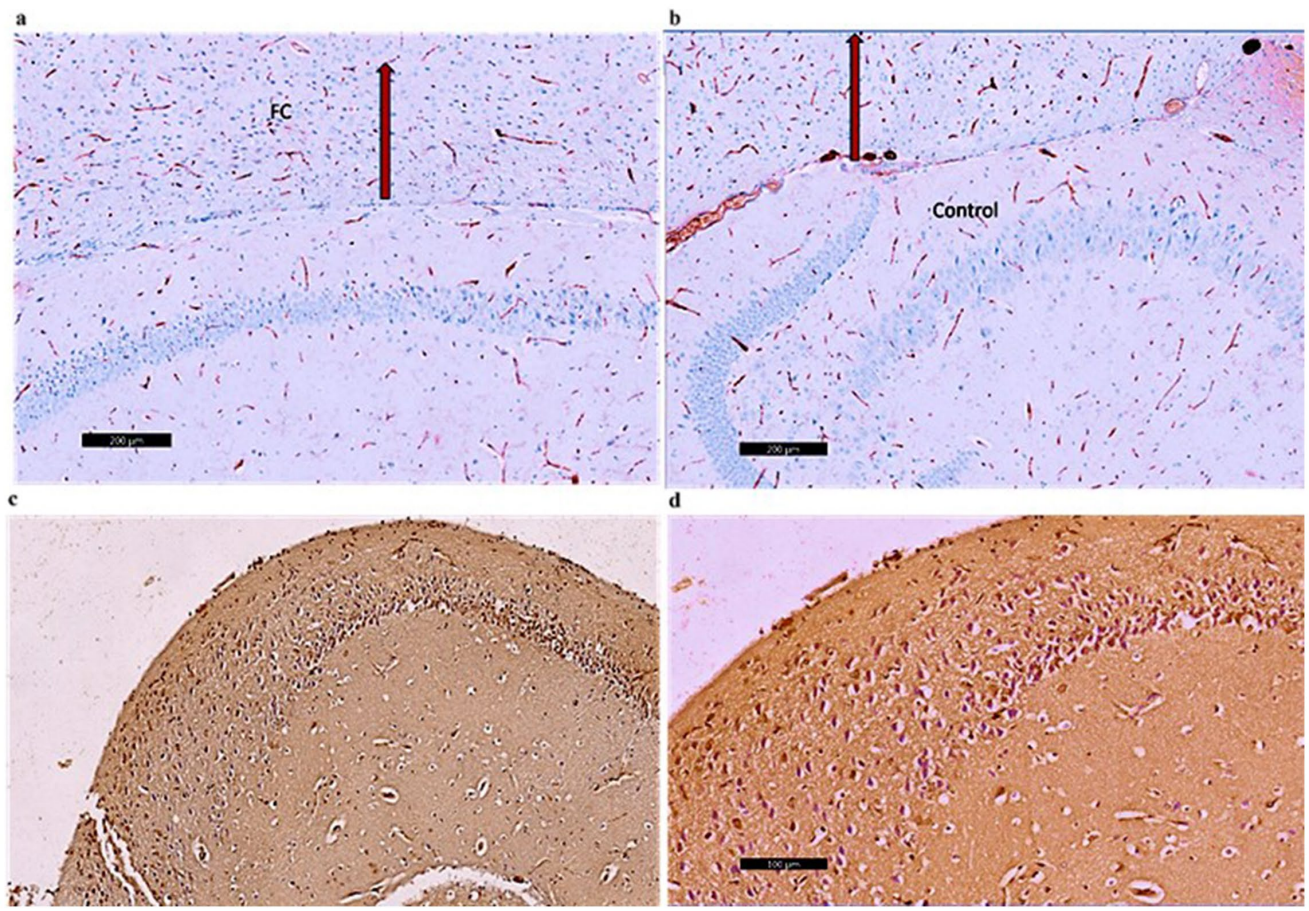
**(a)** A photomicrograph of sections of the control –ve group of adult male SWR Swiss mice showed the normal architecture of the frontal cortex (FC) exhibiting CD4 Mild immunoreaction of the nerve fibers, CD4 ×40. **(b)** A photomicrograph of the *Palmaria p.* group showed the normal architecture of the frontal cortex (FC) exhibiting CD4 mild immunoreaction of the nerve fibers, D4 ×40. **(c)** A photomicrograph of CPZ of the adult male SWR group showed the frontal cortex (FC) exhibiting CD4-marked immunoreaction of the nerve fibers, CD4 ×40. **(d)** A photomicrograph of the *Palmaria p.* + CPZ group showed the frontal cortex (FC) exhibiting CD4 moderate immunoreaction of the nerve fibers, CD4 ×40. (n = 7/group).

Photomicrographs of frontal cortex sections from the control groups displayed normal architecture with mild CD4 immunoreactivity in the nerve fibers (Figures 10a,b). In the CPZ group, the frontal cortex maintained its normal architecture but showed marked CD4 immunoreactivity, indicating an increased immune response (Figure 10c). The CPZ + *Palmaria p.* group exhibited normal frontal cortex architecture with moderate CD4 immunoreactivity in the nerve fibers, suggesting a partial reduction in the immune response compared to the affected group (Figure 10d).

## 4 Discussion

In the current study, *Palmaria p.* exhibited a notable antioxidant capacity, potentially playing a role in its neuroprotective properties. The results of the behavioral studies indicated that CPZ-treated mice exhibited poor episodic memory, as demonstrated by the NORT results. However, the administration of *Palmaria p.* revealed a tendency toward improvements in these deficits. Furthermore, no statistically significant variations were observed in spatial memory and anxiety-related behaviors across the various groups. The results of the gene expression study revealed that CPZ enhanced the expression of CREB and Iba1 in both the hippocampus and frontal cortex. Conversely, *Palmaria p.* exhibited a notable downregulation of CREB, particularly in the hippocampus, and a moderate increase in Iba1 expression. Histopathological studies demonstrated that CPZ elicited structural impairment in the hippocampus and frontal cortex, characterized by diminished cellular dimensions, separation from adjacent tissue, and vacuolations. The administration of *Palmaria p.* mitigated these effects, partially preserving cellular architecture. Immunohistochemical analysis revealed that *Palmaria p.* exhibited a decrease in CD4^+^ cell expression in both areas, indicating a potential mitigation of the immunological response generated by CPZ. Overall, the results highlight the potential of *Palmaria p.* to mitigate cognitive impairments and neuronal damage linked to schizophrenia. The elevated antioxidant capacity of *Palmaria p.*, as demonstrated by the DPPH assay, likely enhances its neuroprotective properties by neutralizing reactive oxygen species and mitigating oxidative damage in the brain, factors recognized as contributors to cognitive deficits in schizophrenia.

Myelination has an impact on processing speed and strength, as well as signal propagation. As a result, it is not surprising that the growth of various cognitive processes corresponds to the myelination of brain regions during development. Cognitive skills such as executive decision-making and impulse control are linked to the development of the prefrontal cortex (PFC) and myelination (Herring and Konradi, 2011). The CPZ-induced schizophrenia model showed specific tract affection as documented in previous literature. The corpus callosum, external capsule, caudate, putamen, and dorsal hippocampal commissure showed obvious demyelination, whereas other tracts appeared to be spared. The cerebral cortex and hippocampus revealed myelin breakdown, while it was modest in the thalamus and hypothalamus. Although the degree of oligodendrocyte (OLs) loss was not consistent across locations, the quantity of OLs was reduced in all of the above regions of CPZ-treated mice (Xu et al., 2010). The aforementioned data are in line with our study, as we found that CPZ induced structural impairment in the hippocampus and frontal cortex.

A meta-analysis indicates that individuals diagnosed with schizophrenia exhibit elevated levels of CD3 + and CD4 + cells, together with an augmented CD4/CD8 ratio. Heightened T cell concentrations in cerebrospinal fluid (CSF) and hippocampus were detected (Ermakov et al., 2022). A study examined microglia, perivascular macrophages, and T-lymphocytes in post-mortem dorsal prefrontal brain tissue from schizophrenia patients and controls to determine immunological dysregulation. Increasing expression of microglial Fcγ receptor CD64 was seen in schizophrenia patients with active psychotic symptoms at death. Psychosis patients had age-dependent increases in Iba1 expression and CD64/HLA-DR ratios, indicating primed/reactive microglia. T-lymphocytes (CD4) may play a role. However, macrophages were not recruited. These findings imply that schizophrenia alters the brain’s immunological environment, depending on symptom status and age (De Picker et al., 2021). A meta-analysis of post-mortem studies on microglia in schizophrenia found an increase in microglial density in various brain areas, with considerable heterogeneity between investigations (van Kesteren et al., 2017). Studies have found activated microglia in the frontal cortex and hippocampus of individuals with late-onset schizophrenia. However, no differences in HLA-DR-labeled microglial density were found in various brain areas, except for a highly elevated density in suicide victims with schizophrenia. Current reports on microglia in schizophrenia patients are still to be elucidated (Petrasch-Parwez et al., 2020; Hercher et al., 2014).

Collectively, the previous findings are concordant with ours in the current study, highlighting the negative impact of CPZ and the mitigation of this effect by *Palmaria p.* alga. In the current study, we assessed the microglial function via the Iba1 gene. Our results are in line with some of the previous reports concerning the CPZ effect. The diminished Iba1 expression in the CPZ + *Palmaria p.* group as compared to the CPZ group signifies attenuated microglial activation. This is consistent with the anti-inflammatory characteristics of *Palmaria p*. and emphasizes its possible function in alleviating neuroinflammatory reactions linked to schizophrenia. We suggest that the contradictory results concerning the microglial function may be due to the stage of schizophrenia during the study, the different experimental models, and the different species. Therefore, our study could shed light on some findings that need more thorough investigation.

The results of the current study indicate that CPZ therapy lowers CREB mRNA levels in the hippocampus and frontal cortex. However, *Palmaria p.*, whether alone or with CPZ, increased expression levels, with the greatest effects when given alone. *Palmaria p.* may preserve or restore gene expression, which might be significant in neuroprotection or neuroregeneration. The noted elevation in CREB expression in the CPZ-treated group may indicate a compensatory response to mitigate CPZ-induced neurotoxicity. The substantial decrease in CREB levels with *Palmaria palmata* co-treatment indicates a restoration of neuroplasticity-associated signaling, perhaps enhancing cognitive results. In concordance with our study, a previous article documented that dopamine, a brain neurotransmitter, is linked to schizophrenia due to hyperactive dopaminergic signal transduction. The cAMP-response element binding protein (CREB) regulates gene expression in dopaminergic neurons. Dopamine affects CREB phosphorylation via G protein-coupled receptors. CREB is a major regulator of neurotrophin responses, and susceptibility genes associated with schizophrenia target and stimulate its activity. Abnormalities in CREB expression are observed in schizophrenic patients (Wang et al., 2018). Another study reported that the mRNA expression of CREB1 was significantly downregulated in psychiatric patients compared to healthy controls. The protein–protein interaction analyses showed that CREB1 directly interacted with several risk genes of psychiatric disorders (Xiao et al., 2018).

Protein synthesis is crucial for long-term memory formation, and CREB/CRE-mediated transcription plays a role in this process. Studies have shown that CREB is involved in various memory processes, including cued and contextual fear memory, spatial memory, olfactory memory, conditioned taste aversion memory, and object and social recognition memory. CREB alpha/delta knockout mice have shown impaired memory formation in context fear conditioning, the Morris water maze, and socially transmitted food preferences. CREB is also involved in cued and contextual fear memory, spatial memory, olfactory memory, conditioned taste aversion memory, and object and social recognition memory (Sakamoto et al., 2011; Sen, 2018; Kogan et al., 1997; Lamprecht et al., 1997). In the current study, we found that NOR is protected by *Palmaria p.*, whilst this effect was not apparent in the spatial memory. Based on previous reports, the PFC has a crucial role in mediating cognitive functions, such as NOR, suggesting that targeting the PFC could enhance cognitive performance in related tasks (Watson et al., 2012). In accordance with these data, we found that CREB expression was upregulated in the *Palmaria palmata*-treated group. These findings support the protective role of *Palmaria p.* on recognition memory and shed light on the involvement of the CREB gene, especially in the frontal cortex, in this process.

In some aspects, mouse novel object recognition is similar to human declarative (episodic) memory, one of the seven aspects of cognition that is impaired in schizophrenia. The most common cause of poor functional outcomes in schizophrenia is cognitive impairment, with psychosis and negative symptoms accounting for the majority of the rest (Rajagopal et al., 2014). In the current study, we observed a significant impairment in NOR memory in the schizophrenia-induced mice (treated with CPZ). Such memory impairment was documented earlier in other studies that used CPZ to induce schizophrenia. For example, it has been documented that the CPZ-induced abnormalities resemble some of the symptoms of schizophrenia. However, they are unlikely to be caused by injury to the entire brain or a particular white matter tract/brain area. However, specific areas seem to be affected, such as the dorsal hippocampus and cerebral cortex (Xu et al., 2010). In the present study, an improvement in the NOR was observed in the *Palmaria p.-*treated group. The latter points to the neuroprotective potential of *Palmaria p.* on the brain structures involved in object recognition memory.

In the present study, we did not note any significant changes in the animal behavior related to spatial memory (assessed by Y maze) and anxiety measures. Earlier studies have reported that the frequency of entries to the arms was not different from that of the control. However, the number of alterations has decreased in the CPZ-induced model of schizophrenia, suggesting impaired memory performance (Xu et al., 2010). In support of this suggestion is what *Garcia and Esquivel* have reported regarding the performance of two mouse strains in the Y-maze in response to stress. They found that the number of arm alternations and incorrect entries in BALB/c mice was substantially higher than in C57BL/6 mice (Garcia and Esquivel, 2018).

Overall, our study highlights the potential of *Palmaria palmata* as a neuroprotective agent capable of mitigating CPZ-induced cognitive deficits and neuroinflammatory responses. Its antioxidant, anti-inflammatory, and gene-modulating properties, particularly involving CREB and Iba1, provide a strong rationale for further exploration in schizophrenia models. However, the current study has some limitations that necessitate further research to investigate the selective impact on specific cognitive domains. Moreover, further in-depth exploration of the mechanistic clues is warranted to fully delineate *the Palmaria p.* therapeutic potential. Additionally, further studies using larger numbers of animals for longer durations may be needed before generalizing the results.

## 5 Conclusion

*Palmaria palmata* alleviates cognitive impairments and neurodegenerative alterations induced by CPZ in a mouse model of schizophrenia. It tends to enhance object recognition memory and promotes neuroplasticity, as demonstrated by elevated CREB expression while also reducing neuroinflammation through the modulation of Iba1 expression. Histological analysis shows that *Palmaria p.* preserves hippocampal and cortical architectures, reduces dentate gyrus thickness, and enhances cellular morphology. It exerts limited influence on anxiety-related behaviors. The neuroprotective and cognitive-enhancing properties of *Palmaria p.* indicate its potential as a natural therapeutic agent for mitigating cognitive deficits associated with schizophrenia. Its antioxidant and anti-inflammatory properties support its suitability as a safer alternative to traditional treatments. Future research should examine the long-term effects of *Palmaria p.* in schizophrenia models and explore its interactions with conventional antipsychotic therapies. Additionally, extended studies involving multiple dosing regimens are required to further assess its anxiolytic and cognitive effects. Clinical trials are also required to confirm its efficacy and safety in human populations.

## Data Availability

The original contributions presented in the study are included in the article/supplementary material, further inquiries can be directed to the corresponding author.

## Footnotes

^1^ https://alborgdigitalpathology.com/Login/Login.aspx
